# Single-gene FISH maps and major chromosomal rearrangements in *Elymus sibiricus* and *E. nutans*

**DOI:** 10.1186/s12870-023-04110-4

**Published:** 2023-02-17

**Authors:** Bo Liu, Jie Chen, Ying Yang, Wenjie Shen, Jialei Guo, Quanwen Dou

**Affiliations:** 1 Key Laboratory of Adaptation and Evolution of Plateau Biota, Northwest Institute of Plateau Biology, Chinese Academy of Sciences, Xining, 810008 Qinghai China; 2 University of Chinese Academy of Sciences, Beijing, 101408 China; 3 Qinghai Provincial Key Laboratory of Crop Molecular Breeding, Northwest Institute of Plateau Biology, Chinese Academy of Sciences, Xining, 810008 Qinghai China

**Keywords:** Chromosome rearrangement, *Elymus sibiricus*, *Elymus nutans*, Inversion, Single-gene FISH

## Abstract

**Background:**

Chromosomal variations have been revealed in both *E. sibiricus* and *E. nutans*, but chromosomal structural variations, such as intra-genome translocations and inversions, are still not recognized due to the cytological limitations of previous studies. Furthermore, the syntenic relationship between both species and wheat chromosomes remains unknown.

**Results:**

Fifty-nine single-gene fluorescence in situ hybridization (FISH) probes, including 22 single-gene probes previously mapped on wheat chromosomes and other newly developed probes from the cDNA of *Elymus* species, were used to characterize the chromosome homoeologous relationship and collinearity of both *E. sibiricus* and *E. nutans* with those of wheat. Eight species-specific chromosomal rearrangements (CRs) were exclusively identified in *E. sibiricus*, including five pericentric inversions in 1H, 2H, 3H, 6H and 2St; one possible pericentric inversion in 5St; one paracentric inversion in 4St; and one reciprocal 4H/6H translocation. Five species-specific CRs were identified in *E. nutans*, including one possible pericentric inversion in 2Y, three possible pericentric multiple-inversions in 1H, 2H and 4Y, and one reciprocal 4Y/5Y translocation. Polymorphic CRs were detected in three of the six materials in *E. sibiricus*, which were mainly represented by inter-genomic translocations. More polymorphic CRs were identified in *E. nutans*, including duplication and insertion, deletion, pericentric inversion, paracentric inversion, and intra- or inter-genomic translocation in different chromosomes.

**Conclusions:**

The study first identified the cross-species homoeology and the syntenic relationship between *E. sibiricus*, *E. nutans* and wheat chromosomes. There are distinct different species-specific CRs between *E. sibiricus* and *E. nutans*, which may be due to their different polyploidy processes. The frequencies of intra-species polymorphic CRs in *E. nutans* were higher than that in *E. sibiricus*. To conclude, the results provide new insights into genome structure and evolution and will facilitate the utilization of germplasm diversity in both *E. sibiricus* and *E. nutans*.

**Supplementary Information:**

The online version contains supplementary material available at 10.1186/s12870-023-04110-4.

## Background

Chromosomal rearrangements (CRs), particularly translocation and inversion, play important roles in karyotype evolution, adaptation divergence, and speciation [1–3]. Species-specific CRs have been frequently observed in Triticeae species [4–6]. Species-specific CRs have been suggested to play an important role in the evolution of these species [7]. Adaptation associated CRs have also been detected by cytology in Triticum species [8]. It has been suggested that adaptive genes can be held together in chromosomal regions with structural variations via recombination suppression, particularly in inversions [2, 9].

*Elymus sibiricus* L. (Siberian wild rye) and *Elymus nutans* Griseb. are two well-known perennial and caespitose grasses belonging to the genus *Elymus* L. in the tribe Triticeae of the family Poaceae, which comprises approximately 150 perennial and exclusively polyploid species [10]. *E. sibiricus* is widely distributed in the northern hemisphere, with a particular preponderance in Sweden, northern Asia, Japan, and North America [11]. It is widely utilized as a forage crop [12, 13]. *E. nutans* is widely distributed in Central and Eastern Asia and the Himalayas [14–16]. Both *E. sibiricus* and *E. nutans* are mostly utilized as forage crops or for grassland restoration because of their high yield, high nutritional value, hardness tolerance, and easy cultivation on the Qinghai-Tibet Plateau (QTP). The developed *E. sibiricus* cultivars Qingmu No. 1, Tongde, Chuancao No.2, Chuancao No.3, and others are widely cultivated in the QTP, and the cultivar Nongmu No.1 is used in Inner Mongolia in China. Furthermore, as the tertiary gene bank for wheat crop development, the high hardness tolerance of *Elymus* species in the QTP has not been explored.

*E. sibiricus* is an allotetraploid species with a genome constitution of StStHH (2*n* = 4*x* = 28) [17], whereas *E. nutans* is an allohexaploid with a genome constitution of StStHHYY (2*n* = 6*x* = 42) [15]. The basic genomes St, H, and Y in *Elymus* are derived from *Pseudoroegneria* (Nevski) Love, *Hordeum* L., and an unknown ancestral species, respectively [18]. Each chromosome of *E. sibiricus* can be clearly distinguished by fluorescence in situ hybridization (FISH) using tandem repetitive sequences such as (AAG)_10_, pAs1, and pSc 119.2 as chromosomal markers [19]. Chromosome polymorphisms in intra- and inter-populations have been revealed by FISH in different *E. sibiricus* accessions. Chromosomes of *E. nutans* can be well characterized by FISH with repetitive sequences similar to those of *E. sibiricus* [20, 21]. The FISH patterns of *E. nutans* were far variated in domesticated or natural populations, those of which exhibited as high frequencies of inter-genomic translocations, amplification, and deletion of repeats [20, 21]. On the other hand, chromosomal variations in *E. nutans* can be indicated by pollen meiosis abnormalities observed in low-fertility plants [22, 23]. Although chromosomal variations were revealed in both *E. sibiricus* and *E. nutans*, chromosomal structural variations, such as intra-genome translocations and inversions, are still not recognized due to the cytological limitations of previous studies. Moreover, the genome of *Elymus* species remains poorly understood. As the species is distantly related to wheat, available genomic information from wheat is very helpful for genetic analysis in both *E. sibiricus* and *E. nutans*. However, the syntenic relationship between both species and wheat chromosomes remains unknown.

Genic sequences are conserved in grass genomes [24]. Chromosomal rearrangements and cross-species homoeology can be investigated by visualizing single-genes on mitotic metaphase chromosomes using FISH [25]. A wheat single-gene FISH map was developed using wheat cDNA sequences as FISH probes [26]. Homoeologous relationships and chromosomal rearrangements have been successfully analyzed in several Triticeae species, such as *Aegilops markgrafii* and *Agropyron cristatum*, using wheat single-gene FISH probes [26–28]. In this study, single-gene FISH mapping was applied to study the chromosome macrostructure, cross-species homoeology, and genome variations in *E. sibiricus* and *E. nutans*.

## Results

### Development of single-gene probes

To confirm the collinearity of the probes, we first electronically hybridized the probe sequences to the genomes of barley and wheat. All single-gene sequences were singly mapped in each subgenome chromosome, except that 7H244 was mapped on both 5L and 7S in wheat chromosomes, in accordance with the two sites detected in barley chromosomes (Table S1). Most of these single-genes were collinear on homoeologous chromosomes of barley and wheat, with a few exceptions. The single-gene 4H604 was mapped to 5AL, 4BL, and 4DL, and 5H587 was mapped to 7BS, 5BL, and 5DL in the wheat chromosomes. The re-circle translocation involving chromosomes 4A, 5A, and 7B in wheat has been reported in many studies [4, 27, 29–31]. This indicates that two new single-genes, 4H604 and 5H587, were involved in the re-circle translocation of 4A-5A-7B. The positions of the new single-gene 4H289 (XM_045125435.1) and 4H242 (XM_045125358.1) localized in the central region of chromosome 4H of barley were opposite to that of the D genome, suggesting the existence of small central region inversions in barley, as reported by Qi et al. [6]. The newly developed single-genes were mapped in the distorted collinearity region, confirming the utility of the probes in detecting chromosomal rearrangements in the Triticeae genomes. Based on the mapping results of the single-genes in the barley and wheat genomes, we found no rearrangement in the D genome, which was also consistent with previous reports [27]. Thus, the chromosomal map of the single-genes in the D genome was used as the standard when studying the homoeology and collinearity of *E. sibiricus* and *E. nutans* chromosomes with other Triticeae species.

Forty-one new cDNA sequences were labeled to hybridize the chromosomes of *H. bogdanii*. Each tested single-gene probe produced a unique site, except for probe 7H244 and 1H514, which showed double sites in different homoeologous group chromosomes. Four probes whose sequences were obtained by cDNA library screening were discarded because they were too close to the other probes to be discriminated from each other by the FISH pattern (Table S1). Together with the 22 single-gene probes previously reported [26], a total of 59 single-gene probes were developed in this study. Seven to ten single-gene probes per chromosome and three to six single-gene probes per arm were used.

### Chromosome structure and major CRs in *H. bogdanii*

**Fig. 1:**
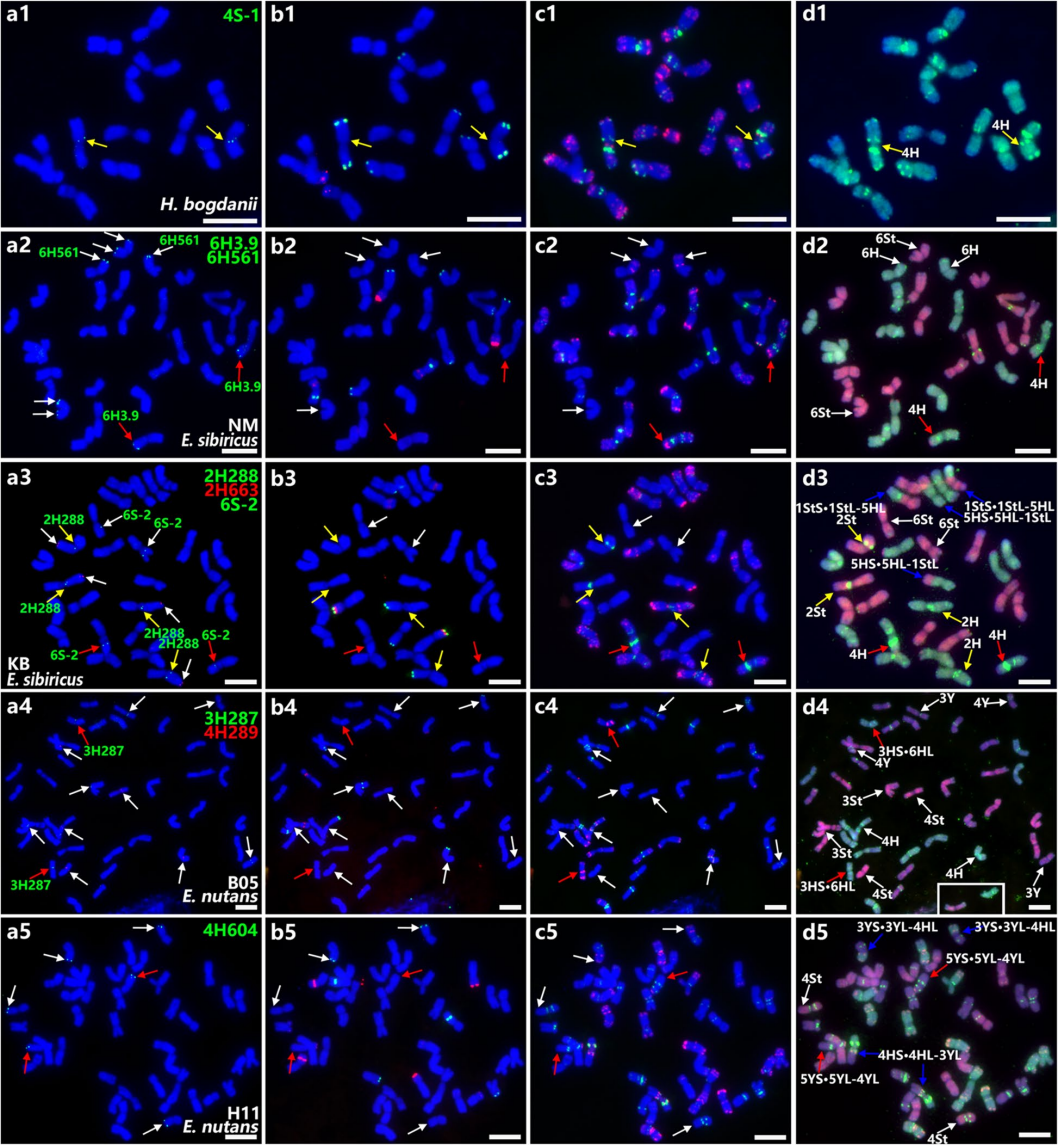
Sequential FISH on mitotic chromosomes of three species with single-gene and repetitive sequence probes. The single-gene probes and their colors are indicated in the top-right corner of the image, and the different species representatives are also illustrated in the plates. **a** single-gene probes (white arrow: single-gene probes hybridized at the expected sites; yellow arrow: single-gene probes hybridized on the expected homoeologous group but unexpected sites; red arrow: single-gene probe hybridized at the non-homoeologous group); **b** 45S rDNA (red) and pSc119.2 (green); **c** pAs1 (red) and (AAG)_10_ (green); **d** genomic DNA probes of *P. stipifolia* (red) and *H. bogdanii* (green) (blue arrows: chromosomes with inter-genomic translocations detected by GISH). Bars = 10 μm

**Fig. 2:**
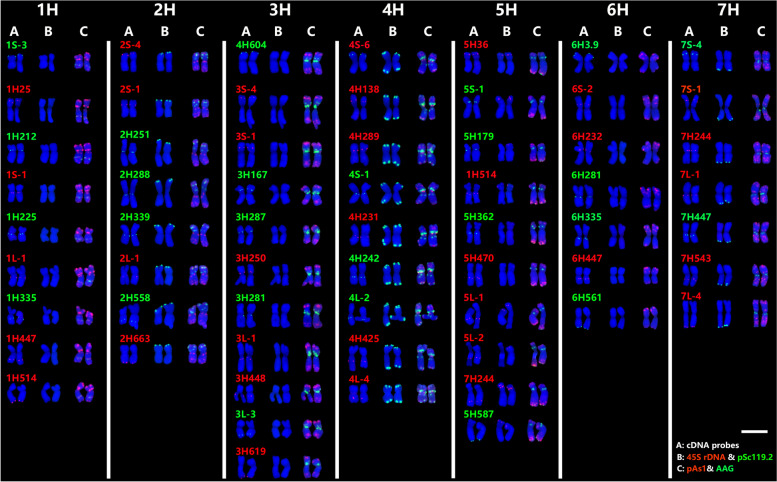
Molecular karyotype of *H. bogdanii* with 59 single-gene probes and repetitive sequences probes. **A** single-gene probes; **B** 45S rDNA (red) and pSc119.2 (green); **C** pAs1 (red) and (AAG)_10_ (green). Bar = 10 μm

**Fig. 3:**
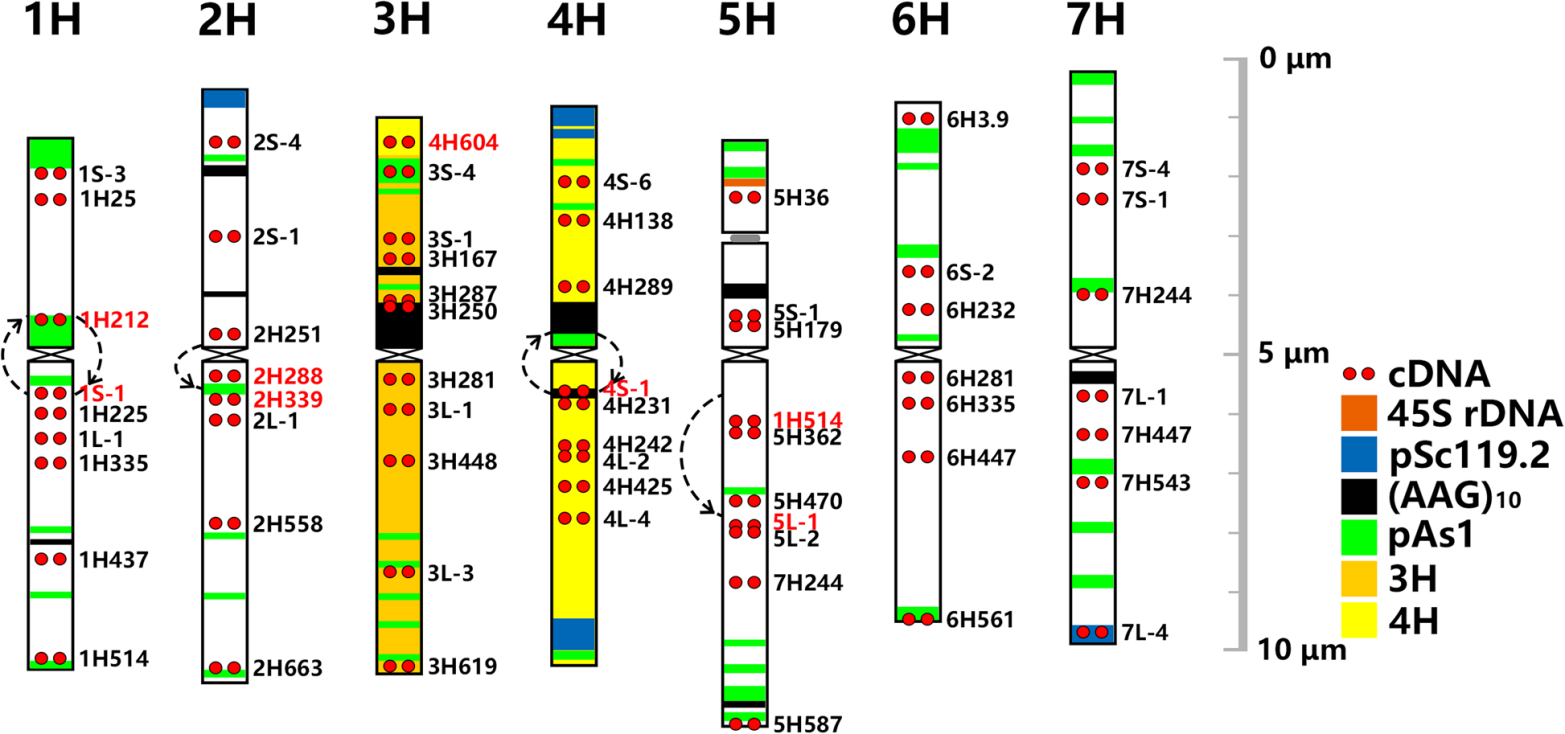
Idiogram for chromosomes of *H. bogdanii* showing the distribution of 45S rDNA, pSc119.2, pAs1, (AAG)_10_ and 59 single-gene probes. The names of single-gene probes that hybridized to the unexpected positions are highlighted in red. Circular arrows and semicircles indicate inversions and collinearity distortion, respectively

*H. bogdanii* is a diploid species and is believed to be an ancestor of the H genome, commonly shared by both *E. sibiricus* and *E. nutans*. *H. bogdanii* chromosomes can be clearly distinguished from each other using combinations of a few repetitive sequences [32]. In the present study, each chromosome was characterized by combination of probes of pSc119.2, 45S rDNA, pAs1, and (AAG)_10_. To test the efficiency of the developed single-gene probes and track possible CRs in *E. sibiricus* and *E. nutans,* we hybridized 59 single-gene probes to the chromosomes of *H. bogdanii* (Fig. 1a1-d1; Fig. 2). Fifty-one of the 59 single-gene probes were physically mapped exclusively to the expected chromosomal positions, with eight exceptions representing major CRs (Fig. 3; Table S2). This indicates that the collinearity of *H. bogdanii* chromosomes with those of wheat or barley could be exactly determined. The collinearity of 6H and 7H were wholly maintained the overall length of the chromosomes. The collinearity of 1H was simply interrupted by a pericentric inversion, with broken positions near the centromere in both arms. Chromosome 2H may have involved a pericentric inversion with an interstitial broken position in the short arm, another broken position near the centromere in the long arm, and a secondary paracentric inversion in the long arm (Fig. S1a, PeI I and PaI I). Chromosome 3H was translocated by a distal segment from 4HL to the distal part of the short arm. Chromosome 4H may contained a simple pericentric inversion and distal deletion in the long arm (Fig. S2a, PeI I). The arrangement of the long arm of 5H was more complex, which may include two paracentric inversions involving probes 5L-1, 5H362, and 5H470 (Fig. S2b, PaI I and PaI II), and another intercalary duplication and insertion segment from the distal part of the long arm of 1H (Fig. 3).

### Chromosome structure and major CRs in *E. sibiricus*

**Fig. 4:**
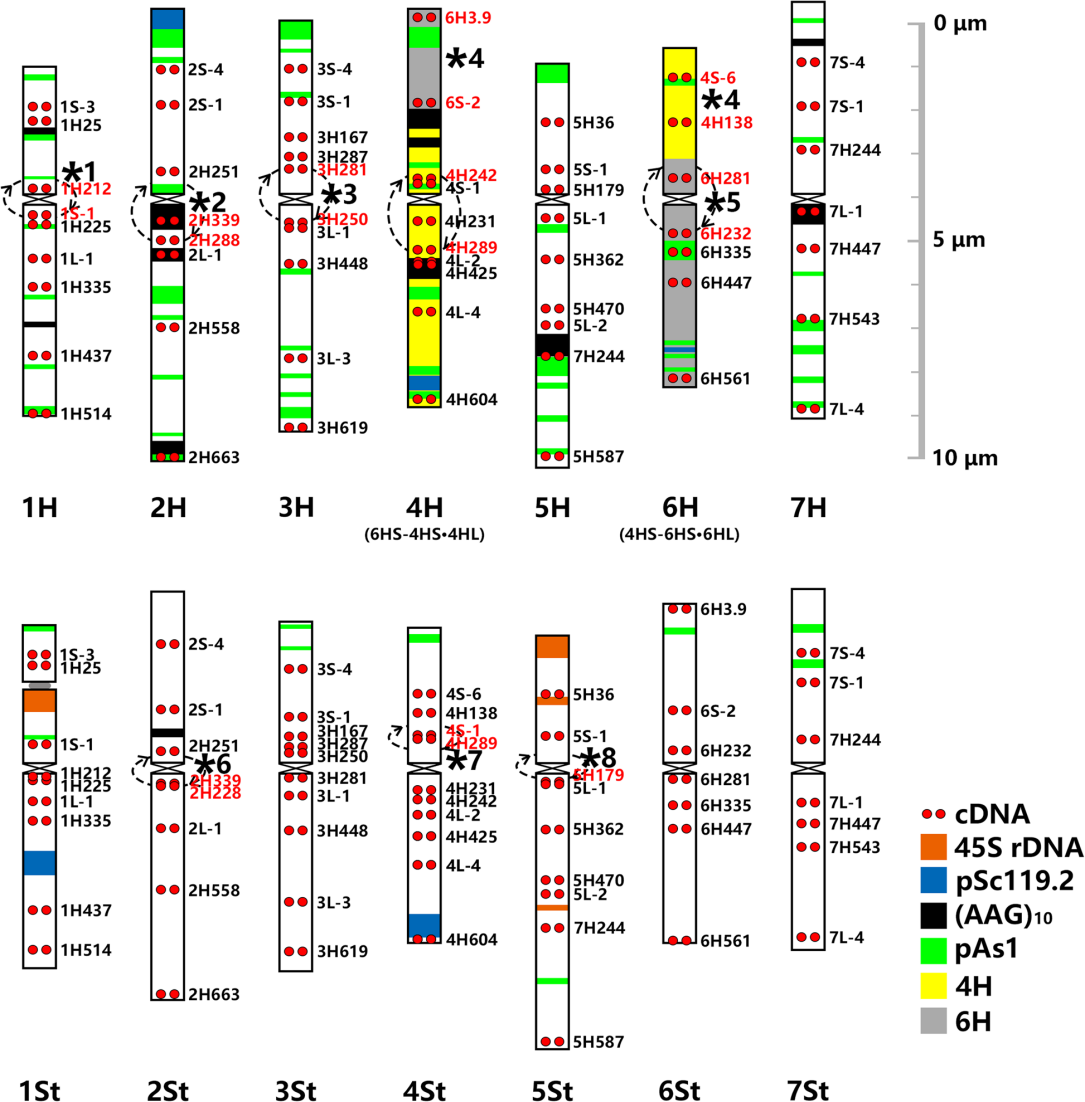
Idiogram for chromosomes in material DY of *E. sibiricus* showing the distribution of 45S rDNA, pSc119.2, pAs1, (AAG)_10_ and 59 single-gene probes. The names of single-gene probes that hybridized to the unexpected positions are highlighted in red. Inversions are indicated by circular arrows. Eight species-specific CRs are indicated by numbered “ * ”

Each chromosome of *E. sibiricus* can be identified using FISH probes of tandem repeats, including pAs1, pSc119.2, (AAG)_10_, 5S, and 45S rDNA, in a previous study [19]. Further, FISH mapping with 59 single-gene probes allowed the macrostructure and homoeology of the *E. sibiricus* chromosome to be compared to wheat or barley (Fig. 1a2-d3; Fig. S3; Table S3). The chromosomes featured by repetitive sequence markers with pSc119.2, 45S rDNA, pAs1, and (AAG)_10_, and were re-designated according to their homoeology to wheat. Conserved major chromosome CRs were identified in six materials, but accidental polymorphic CRs were identified in three materials (Fig. 4; Fig. S4). Eight species-specific CRs were exclusively identified in *E. sibiricus*, including five pericentric inversions in 1H, 2H, 3H, 6H and 2St; one possible pericentric inversion in 5St; one paracentric inversion in 4St; and one reciprocal 4H/6H translocation (Fig. 4). Chromosome collinearity was maintained throughout the entire chromosome length on 5H, 7H, 1St, 3St, 6St, and 7St compared to that in wheat and barley. In the H genome, 1H, 2H, and 3H were mostly collinear over their entire length to wheat or barley chromosomes, with exceptional pericentric inversions in each chromosome. The pericentric inversion in 1H was similar to that in 1H in *H. bogdanii*, which may be inherited from the H genome progenitor. The inversion in 2H in *E. sibiricus* was different from that in 2H in *H. bogdanii*, with the reverse position of probes 2H339 and 2H288 in the long arm, and it should have experienced only one pericentric inversion from its ancestor (Fig. S1a, PeI I). Chromosome 4H was distinctly rearranged because of twice pericentric inversion (Fig. S2a, PeI II and PeI IV) in all tested materials, except XN. Reciprocal translocations were found at the distal part of the short arms of chromosomes 4H and 6H, and chromosome 6H was also distinctly rearranged by a pericentric inversion (Fig. 4; Fig. S2a, PeI). In the St genome, chromosomes 2St, 4St, and 5St were mostly collinear over their entire length to wheat or barley chromosomes but rearranged with a pericentric inversion (Fig. S1a, PeI I), a paracentric inversion (Fig. S2a, PaI II), and a possible pericentric inversion (Fig. S2b, PeI I), respectively (Fig. 4; Table 1).

**Table 1:** Chromosomal structure variation in 11 materials of three species

Chromosome	Chromosomal structure variation	*H. bogdonii*	*E. sibiricus*						*E. nutans*			
			DY	GEM	KB	XN	NM	LJS	PJC	B05	H11	G04
1H	PeI I	+ ’	+ ’	+ ’	+ ’	+ ’	+ ’	+ ’	+ ’’	+ ’’	+ ’’	+ ’’
	PeI II	-	-	-	-	-	-	-	+ ’	+ ’	+ ’	+ ’
1St	RT (5H/1St)	0	-	-	+	-	-	-	-	-	-	-
	De (1H514)	0	-	-	-	-	-	-	+	-	-	-
1Y	Du (2H558)	-	-	-	-	-	-	-	-	-	-	+
2H	PaI I	+	-	-	-	-	-	-	-	-	-	-
	PeI I	+ ’’	+ ’	+ ’	+ ’	+ ’	+ ’	+ ’	+ ’’	+ ’’	+ ’’	+ ’’
	PeI II	-	-	-	-	-	-	-	+ ’	+ ’	+ ’	+ ’
	RT (2H/2St)	-	-	-	-	-	-	+	-	-	-	-
2St	PeI I	0	+ ’	+ ’	+ ’	+ ’	+ ’	+ ’	-	-	-	-
	RT (2H/2St)	0	-	-	-	-	-	+	-	-	-	-
	RT (4H/2St)	0	-	-	-	-	-	-	-	-	-	+
2Y	PeI III	0	0	0	0	0	0	0	+ ’	+ ’	+ ’	+ ’
3H	Du (3H448)	-	-	-	-	-	-	-	+	+	-	+
	T (4H)	+	-	-	-	-	-	-	-	-	-	-
	RT (3H/6H)	-	-	-	-	-	-	-	-	+	-	-
	PeI	-	+ ’	+ ’	+ ’	+ ’	+ ’	+ ’	-	-	-	-
3St	Du (3H448)	-	-	-	-	-	-	-	-	+	+	+
3Y	De (3H287)	0	0	0	0	0	0	0	+	-	+	-
	PaI, RT (4H/3Y)	0	0	0	0	0	0	0	-	-	+	-
4H	Du (2L-1)	-	-	-	-	-	-	-	-	+	-	+
	PeI I	+	-	-	-	-	-	-	+	-	-	-
	PeI II	-	+ ’’	+ ’’	+ ’’	+ ’’	+ ’’	+ ’’	-	-	-	-
	PeI III	-	-	-	-	+	-	-	-	-	-	-
	PeI IV	-	+	+	+	-	+	+	-	-	-	-
	PeI V	-	-	-	-	-	-	-	-	-	-	+
	PaI I	-	-	-	-	-	-	-	+ ’’	+ ’’	+ ’’	+ ’’
	RT (4H/6H)	-	+ ’	+ ’	+ ’	+ ’	+ ’	+ ’	-	-	-	-
	RT (4H/3Y)	-	-	-	-	-	-	-	-	-	+	-
	RT (4H/2St)	-	-	-	-	-	-	-	-	-	-	+
	T (4H)	+	-	-	-	-	-	-	-	-	-	-
4St	PaI II	0	+ ’	+ ’	+ ’	+ ’	+ ’	+ ’	-	-	-	-
4Y	PeI VI, PeI VII, RT (4Y/5Y)	0	0	0	0	0	0	0	+ ’	+ ’	+ ’	+ ’
5H	Du (1H514)	+	-	-	-	-	-	-	-	-	-	-
	PaI I, II	+	-	-	-	-	-	-	-	-	-	-
	RT (5H/1St)	-	-	-	+	-	-	-	-	-	-	-
5St	PeI I	0	+ ’	+ ’	+ ’	+ ’	+ ’	+ ’	-	-	-	-
5Y	PeI I, RT (4Y/5Y)	0	0	0	0	0	0	0	+ ’	+ ’	+ ’	+ ’
	PeI II	0	0	0	0	0	0	0	-	-	+	-
6H	PeI	-	+ ’	+ ’	+ ’	+ ’	+ ’	+ ’	+	-	+	-
	RT (4H/6H)	-	+ ’	+ ’	+ ’	+ ’	+ ’	+ ’	-	-	-	-
	RT (3H/6H)	-	-	-	-	-	-	-	-	+	-	-
7H	PaI	-	-	-	-	-	-	-	-	+	-	+

" + " means the presence of chromosomal structural variation; "-" means that structural variation was absent; "0" means that no such subgenome exists in this species. Species-specific CRs are superscripted by " ’ ", and structural variations that may be derived from a common ancestor are superscripted by " ’’ "

Abbreviation for chromosomal structural variation is as follows: *PaI* paracentric inversion, *PeI* pericentric inversion, *RT* reciprocal translocation, *T* translocation, *Du* duplication and insertion, *De* deletion

Specifically, inter-genomic translocations were detected in the LJS and KB materials. Mapping with single-gene probes revealed that material LJS had a reciprocal translocation between 2H and 2St, whereas a large part of the long arm of 2H was translocated by a small part of the short arm of 2St (2HL-2StS·2StL) and a large part of the short arm of 2St was translocated by a small part of the long arm of 2H (2HS·2HL-2StS; Fig. S4a). Material KB involved a reciprocal translocation between 5H and 1St, whereas the distal parts of the long arm of 5H and 1St were reciprocally translocated (Fig. 1a3-d3; Fig. S4b). The pericentric inversion of 4H in material XN was exceptional compared to that in the others (Fig. S4c Fig S2a PeI III).

### Chromosome structure and major CRs in *E. nutans*

**Fig. 5:**
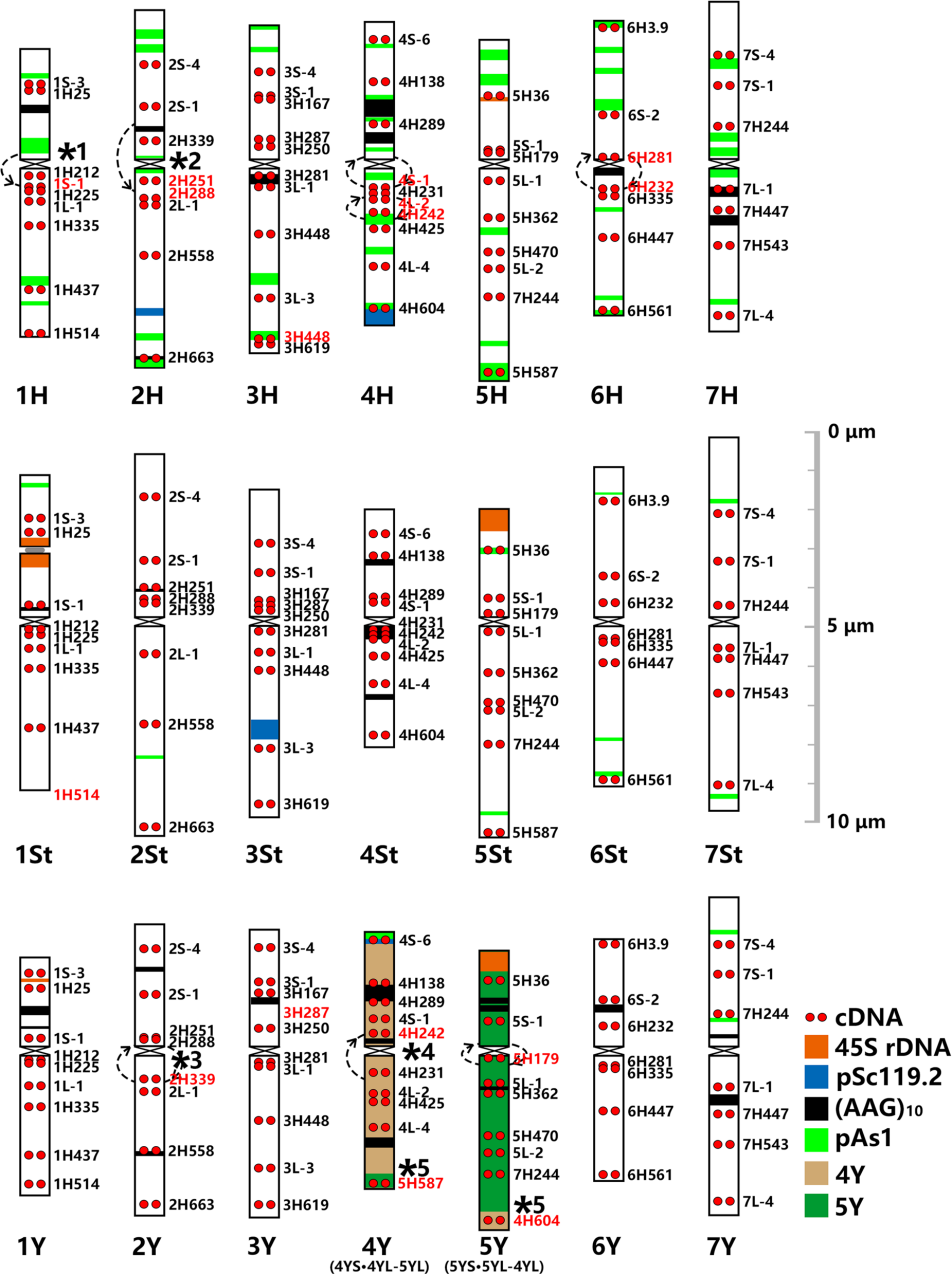
Idiogram for chromosomes in material PJC of *E. nutans* showing the distribution of 45S rDNA, pSc119.2, pAs1, (AAG)_10_ and 59 single-gene probes. The names of single-gene probes that hybridized to the unexpected sites are highlighted in red. The name of probe in the deletion of chromosome 1St is also marked in red. Circular arrows and semicircle indicate inversions and collinearity distortion, respectively. Five species-specific CRs are indicated by numbered “ * ”

High chromosome polymorphism was also detected in *E. nutans*, as described previously [20, 21], but the homoeologous group was entirely determined by single-gene FISH in four different *E. nutans* materials in the present study (Fig. 1 a4-d5; Fig. 5; Fig. S5; Table S4). Different CRs were identified in different materials. However, five conserved species-specific CRs, including 1 possible pericentric inversion in 2Y, three possible pericentric multiple-inversions in 1H, 2H and 4Y, and 1 reciprocal 4Y/5Y translocation were commonly shared by 4 materials (Fig. 5; Table 1). Furthermore, chromosome 4H had the worst collinearity; however, it is possible that all *E. nutans* may have experienced a common paracentric inversion (Fig. S2a, PaI I). The single-gene FISH pattern around centromere in 1H of *E. nutans* strongly implies another inversion accident derived from the pericentric inversions as in 1H of *H. bogdanii* and *E. sibiricus* (Table 1). The pericentric inversion in 2H was different from those in *H. bogdanii* and *E. sibiricus* from the deduced breakage points, but 2H in all three species may have experienced a common pericentric inversion (Fig. S1a, PeI I). Chromosomes 2Y included a possible pericentric inversion with breakage points near the centromere in both arms (Fig. 5; Fig. S1a, PeI III). Chromosomes 4Y may have experienced two successive pericentric inversion (Fig. 5; Fig. S2a, PeI VI and PeI VII). A tentative reciprocal translocation between the distal end of the 4YL and the distal part of the 5YL was observed in all the tested materials. A common proximal possible pericentric inversion in 5Y was shared by 3 materials (Fig. S2b, PeI I). However, the inversion in 5Y of material H11 was mostly derived from the previous inversion type by another inversion event (Fig. S2b, PeI II). Species-specific CRs were not identified in the St genome for any material.

Fifteen types of no species-specific CRs (polymorphic CRs) were identified in the investigated materials of *E. nutans*. Nine polymorphic CRs were material specific and distributed in four different materials with no overlap (Table 1; Fig. S6). Material PJC contained a unique deletion (1H514) at the end of the long arm of chromosome 1St, and a possible pericentric inversion (Fig. S2a, PeI I) near centromere in both arms in 4H; material B05 contained a reciprocal translocation between the most parts of the long arms of 3H and 6H; material H11 contained a paracentric inversion in the long arm near the centromere of 3Y, a reciprocal translocation between the most parts of the long arms of 4H and 3Y and a more complex pericentric inversion in chromosome 5Y (Fig. S6); material G04 contained a duplication and insertion (2H558) in the interstitial region of the long arm of chromosome 1Y, a pericentric inversion linked to another paracentric inversion in chromosome 4H (Fig. S2a, PeI V), and a reciprocal translocation between most parts of the short arm of 4H and the long arm of 2St. Four polymorphic CRs were shared by two of the four materials (Table 1; Fig. S6). Materials PJC and H11 contained a possible deletion (3H287) in chromosome 3Y and a pericentric inversion of chromosome 6H, while materials B05 and G04 contained a duplication and insertion (2L-1) at the interstitial of the short arm of chromosome 4H and a paracentric inversion in the long arm of 7H. One polymorphic CRs of duplication and insertion (3H448) at the end of the long arm of chromosome 3H was shared by three materials, except H11, and another polymorphic CRs of duplication and insertion (3H448) at the end of the long arm of chromosome 3St was shared by three materials, except PJC. (Table 1; Fig. S6).

### Chromosome polymorphisms in intra-species

In addition to the polymorphic CRs detected in different materials in both *E. sibiricus* and *E. nutan*s, chromosome polymorphisms were still identified by FISH patterns of repetitive sequences. Because the homoeologous group of each chromosome was exclusively determined, polymorphism of the homologous chromosomes could be estimated by investigating the limited materials in each species.

In *E. sibiricus,* a total of 32 chromosome variants were identified across four different materials, whereas 19 and 13 variants were identified in the H and St genomes, respectively. Chromosomes 3H, 5H, and 6H were the most conserved, with no more than two variants each, whereas 1H, 2H, and 4H were the most variable, with four variants each in the H genome. Chromosomes 3St, 4St, 6St, and 7St were the most conserved with only one variant, whereas 2St was the most variable with four variants in the St genome (Table 2; Fig. S7). Most of the chromosome variants were due to the presence/absence of the signals of pSc119.2 and hybridization intensity variation of (AAG)_10_, while minor variants were verified as variations with polymorphic CRs.

**Table 2:** Chromosome variants in 6 materials of *E. sibiricus* and 4 materials of *E. nutans*

Species	Materials	H genome							St genome							Y genome						
		1	2	3	4	5	6	7	1	2	3	4	5	6	7	1	2	3	4	5	6	7
*Elymus sibiricus*	DY	a	a	a	a	a	a	a	a	a	a	a	a	a	a	-	-	-	-	-	-	-
	GEM	b	a	a	a	a	a	b	a	a	a	a	a	a	a	-	-	-	-	-	-	-
	KB	a	b	a	b	b’	a	a	b’	a	a	a	b	a	a	-	-	-	-	-	-	-
	XN	c	c	a	a’	a	a	c	a	b	a	a	c	a	a	-	-	-	-	-	-	-
	NM	c	c	a	c	a	a	a	a	c	a	a	c	a	a	-	-	-	-	-	-	-
	LJS	d	d’	a	c	a	a	b	a	d’	a	a	c	a	a	-	-	-	-	-	-	-
No. of variants with polymorphic CRs		0	1	0	1	1	0	0	1	1	0	0	0	0	0							
No. of variants		4	4	1	4	2	1	3	2	4	1	1	3	1	1	-	-	-	-	-	-	-
*Elymus nutans*	Total	19							13							-						
	PJC	a	a	a’	a’	a	a’	a	a’	a	a	a	a	a	a	a	a	a’	a	a	a	a
	B05	a	b	b’	b’	a	b’	b’	b	b	a’	b	b	a	b	b	b	b	b	b	b	b
	H11	b	c	a	c’	a	a’	a	a	c	a’	c	a	b	c	b	c	c’	c	c’	c	c
	G04	b	d	c’	d’	a	c	b’	a	d’	b’	b	a	c	a	a’	b	d	b	d	d	d
No. of variants with polymorphic CRs		0	0	3	4	0	2	1	1	1	2	0	0	0	0	1	0	2	0	1	0	0
No. of variants		2	4	4	4	1	3	2	3	4	3	3	2	3	3	3	3	4	3	4	4	4
	Total	20							21							25						

The same FISH pattern of repetitive sequences is indicated by the same letter, and chromosome variants with additional polymorphic CRs are superscripted by " ’ "

Fewer materials have been investigated in *E. nutans* than in *E. sibiricus.* However, more chromosome variants were identified in each genome of *E. nutans* than in those of *E. sibiricus*. A total of 66 chromosome variants, including 20, 21, and 25 variants in the H, St, and Y genomes, respectively, were detected in 4 materials of *E. nutans*. Chromosomes 1H, 5H, and 7H were the most conserved, with no more than two variants each, whereas 2H, 3H, and 4H were the most variable, with four variants each in the H genome. Chromosome 5St was the most conserved with two variants, whereas 2St was the most variable with four variants in the St genome. Chromosome 1Y, 2Y and 4Y was most conserved with 3 variants, whereas 3Y, 5Y, 6Y and 7Y were most variable with 4 variants each in Y genome (Table 2, Fig. S7). The chromosome variation patterns were similar to those in *E. sibiricus,* mainly attributed to the variation in FISH patterns of pSc119.2 and (AAG)_10_ (Fig. S7). However, high rates of variants (18 of 66) with polymorphic CRs have been identified in *E, nutans*. The H genome had the highest number of variants with polymorphic CRs (10), of which 3H, 4H, 6H, and 7H were 3, 4, 2, and 1, respectively. The St genome had four variants with polymorphic CRs, of which 1St, 2St, and 3St were 1, 1, and 2, respectively. The Y genome also had 4 variants with polymorphic CRs, of which 1Y, 3Y and 5Y were 1, 2 and 1, respectively (Table 1, Fig. S6). The number of variants (18) with polymorphic CRs was higher than the types number (14) of polymorphic CRs because although some materials had the same type of polymorphic CRs, they differed in repetitive FISH patterns.

## Discussion

### Species-specific CRs in three species

In total, eight and five species-specific CRs were identified in six *E. sibiricus* materials and four *E. nutans* materials, respectively. The pericentric inversion in 1H was commonly shared by both *H. bogdanii* and *E. sibiricus*. The CRs around centromere in 1H of *E. nutans* was suggested to be derived by further arrangements from the same ancient pericentric inversion in *H. bogdanii* and *E. sibiricus*. This suggests that the pericentric inversion in 1H of *H. bogdanii* is more ancient and H genome specific. Five other CRs in *H. bogdanii* were not directly repeatably detected in *E. sibiricus* and *E. nutans*, but chromosomes 2H may have experienced a common pericentric inversion (Fig. S1a, PeI I). This implies the possible CRs polymorphism in *H. bogdanii*, different H genome providers, or chromosome structure alteration after polyploidization. The distinctly different CRs patterns between *E. sibiricus* and *E. nutans* are due to their different polyploidy processes, which involved once and twice hybridization events, respectively. The CRs distributed in the St and H chromosomes in *E. sibiricus* reflect the response to polyploidization by both genomes after hybridization between the diploid St and H ancestors. However, species-specific CRs in *E. nutans* were revealed in the Y and H genomes but not in the St genome. The StYH species in *Elymus* were possibly derived from two hybridization events, in which the tetraploid StY genome was first created and then hybridized with the diploid *Hordeum* second [17, 33, 34]. Two hybridization events are believed to be the evolution of common wheat, whose species-specific CRs were observed to be shared with ancestral tetraploid wheat (*Triticum turgidum* L.) [4, 35]. A wide investigation of CRs in StY species may provide important clues regarding the origin of species-specific CRs in StHY species.

Species-specific CRs play an important role in restoring fertility and nucleo-cytoplasmic compatibility for the genetic stabilization of newly formed hybrids and polyploids [7]. The hypothesis of nucleo-cytoplasmic interaction (NCI) was not only supported by species-specific translocation in the form of *Triticum turgidum* and *T. timopheevii* [5, 35], but was also confirmed by fertility restoration in the progeny derived from hybrids between *E. trachycaulus* and common wheat [36] and even in *Nicotiana tabacum* allopolyploids [37]. Gill et al. [38] summarized that species-specific CRs driven by NCI were closely related to the long arm of homoeologous chromosome 4 and the short arm of homoeologous 5 in Triticeae species. In the present study, three species-specific CRs were involved in homoeologous chromosomes 4 and 5 in *E. sibiricus*. In *E. nutans*, homoeologous chromosomes 4 and 5 involve two species-specific CRs, and chromosome 4H may have experienced a common paracentric inversion. In addition, a common possible pericentric inversion in 5Y was shared by 3 materials, and the inversion in 5Y of material H11 was derived from the common inversion.

It has been proposed that allopolyploidization accelerates genome evolution in two ways: through “revolutionary” changes that arise shortly after genomes are merged, versus “evolutionary” changes that occur more gradually over time [39]. Chromosome instability has been reported in resynthesized and recently formed naurtal allopolyploid species [40, 41]. Alteration of gene expression at or near the breakpoints of CRs may cause over-dominance, leading to its selection (fixation) [42]. Other than NCI related CRs were found in *E. sibiricus* and *E. nutans.* If NCI related CRs reflects “revolutionary” changes, the other CRs may represent “evolutionary” changes, which may play roles in maintaining genome stability or selection advantage. However, this requires further elucidation.

### Recurrent use of the CRs breakpoints in Triticeae

The recurrence of CRs and reuse of DNA breakpoints have been mostly demonstrated in Triticeae species [6, 42]. The 4L/5L reciprocal translocation was found in several Triticeae species with identical or very close breakpoint positions [4, 42, 43]. In the present study, the specific reciprocal translocation 4Y/5Y was well identified in *E. nutans* by single-gene FISH. The reciprocal translocation of the broken point in 4Y (Fig. S2c, point G) delimitated by single-gene FISH probes and genome sequence survey in wheat suggests the 4Y/5Y broken points to be collinear with those in other Triticeae species. In addition, a translocation from the distal part of 4HL to 3HS was revealed in *H. bogdanii*. These results suggest a conserved broken point in the distal part of the long arms of the homoeologue group 4 chromosomes. A high rate of pericentric inversions has been observed in group 4 chromosomes of different Triticeae species with different broken points [6]. Multiple pericentric inversions in 4H, 4St, and 4Y were revealed in *E. sibiricus* and *E. nutans*, and high-frequency translocations involving 4H with broken points near the centromere were revealed in *E. nutans* in this study. A few of pericentromeric breakpoints in group 4 were tentatively inferred with 2–5 times independent origins for each breakpoint (Fig. S2c). In this study, we speculated that breakpoint E reuses four times between probes 4H138 (4D pseudomolecule 114.1 M) and 4H289 (4D pseudomolecule 172.0 M) in the short arm of group 4 (Fig. S2c, point E), which delimited the possible conserved breakpoint described in wheat by ESTs [6]. Similar results were found in group 2. Our results provide further evidence of widespread breakpoint reuse in Triticeae species.

### Association between stability of sub-genomes and CRs in *E. nutans*

Our previous study suggested that the St and H genomes may contain more chromosomal structural variations than the Y genome in *E. nutans* [22, 23]. However, the H genome contained a high number of polymorphic CRs, and the St and Y genomes had low and similar frequencies of those in the present study. This indicates that the chromosome structures of the St and Y genomes are more stable than those of the H genome. Based on the characteristics of repeat sequences FISH patterns and polymorphic CRs, it was revealed that the Y genome has the most chromosome variants. Similar results have been found in wild populations based on the characteristics of repeat sequence FISH patters [21]. These results suggest that the meiosis stability of St, H, and Y was not simply determined by variations of the repeat distribution or genome structure of each. The meiotic stability of the heterozygote may be strongly affected by factors other than chromosome structure variability in *E. nutans*. Many genes are involved in making meiosis efficient in plants [44]. Although meiosis is conserved, the proteins that orchestrate meiosis are often surprisingly divergent in the primary sequence and meiosis genes under directional selection in natural populations [45]. The under dominance (low seed set) of heterozygotes of *E. nutans* may be hybrids between diverged populations, and the instability of those meiosis may be strongly affected by incompatible genes related to meiosis. Since the majority of species-specific CRs exist in the Y genome, whether they contribute much to the stability of the Y genome or whether Y genome meiosis is less affected by the alteration of meiosis gene expression, further molecular evidence is required.

### Association between adaptation divergence and CRs

High and low frequencies of intra-species polymorphic CRs were revealed in *E. nutans* and *E. sibiricus*, respectively, which were collected in different environments. CRs, including inversions and translocations, can hold adaptive genes in chromosomal regions via recombination suppression [2, 9, 46]. Introgression hybridization with related species can introduce CRs with divergent blocks in the genome [27, 47, 48]. *E. nutans* exhibits high cross ability with other *Elymus* related taxa [15, 49]. The intra-species polymorphic CRs are highly possible to be introduced from related species by introgression hybridization. Since the CRs regions can be delimited by single-gene FISH mapping and their collinear regions in wheat or barley can be easily matched, genetic divergence between CRs and other genome regions can be uncovered by phylogenetic analysis. Further association analysis between the gene expression patterns of the CRs regions and ecological variance can facilitate the identification of adaptation related genes.

Furthermore, the major CRs revealed in this study will assist in the assembly of the complete genome of *E. sibiricus* and *E. nutans* and will also be helpful in evaluating and utilizing germplasm diversity in both species.

## Conclusions

The present study used a set of single-gene probes, including 37 newly developed ones, first identified the cross-species homoeology and the syntenic relationship between *E. sibiricus*, *E. nutans* and wheat chromosomes. There are distinct different species-specific CRs between *E. sibiricus* and *E. nutans*, which may be due to their different polyploidy processes. The results provide new insights into genome structure and evolution and will facilitate the utilization of germplasm diversity in both *E. sibiricus* and *E. nutans*.

## Methods

### Plant material

The root tips of one material of *Hordeum bogdanii*, six materials of *E. sibiricus*, and four materials of *E. nutans* were used in this study. In addition, the leaves of *Hordeum bogdanii* and *Pseudoroegneria stipifolia*, an St genome donor species, were used in this study (Table 3). All the materials are planted in an experimental plot in Xining, Qinghai.

**Table 3:** Plant materials used in this study

Species	Identification ID	2*n*	Genome	Source
*Pseudoroegneria stipifolia*	PI 313,960	14	St	FRRL, USA
*Hordeum bogdanii*	Hbw-002–3	14	H	Golmud, Qinghai
*Elymus sibiricus*	DY (Cultivar Qingmu No.1)	28	StH	Tongde, Qinghai
	GEM	28	StH	Golmud, Qinghai
	KB	28	StH	Aba, Sichuan
	XN	28	StH	Menyuan, Qinghai
	NM (Cultivar Nongmu No. 1)	28	StH	Inner Mogolia
	LJS	28	StH	Guide, Qinghai
*Elymus nutans*	PJC	42	StYH	Tongde, Qinghai
	B05	42	StYH	Haiyan, Qinghai
	H11	42	StYH	Haiyan, Qinghai
	G04	42	StYH	Gonghe, Qinghai

### Preparation of cDNA sequences

A total of 59 cDNA sequences were used in this study, 22 of which were previously anchored to wheat chromosomes by FISH [26] and kindly provided by the National BioResource Project-Wheat, Japan (https://nbrp.jp/en/). Thirty-seven cDNA sequences were newly developed using two strategies.

In the first strategy, we screened a constructed full-length cDNA library of *Elymus breviaristatus* Keng. Clones more than 2 kb in length were selected and sequenced. Further BLAST against the barley reference genome (MorexV3_pseudomolecules_assembly) and wheat reference genome (IWGSC CS RefSeq v1.0) allowed the identification of the cDNA of the unique site in each homoeologous group. Twelve full-length cDNAs that met these requirements were screened. In the second strategy, 34 single-copy genes with conserved collinearity were predicted and obtained by screening the pseudomolecules of barley and wheat genomes with lengths of more than 2 kb and evenly spanned approximately 100 Mb. The transcript profile of *H. bogdanii* was obtained by PacBio sequencing, and RNA-Seq was performed by Biomarker Technologies Co., Ltd. (Beijing, China). Local BLAST against the transcriptome data of *H. bogdanii* with 34 predicted single-copy genes enabled identification of homologues in *H. bogdanii*. The target cDNA was amplified by PCR using reverse transcribed mRNA of *H. bogdanii* as a template with specific primers. The PCR products were cloned into the pCE2 TA/Blunt-Zero vector (Vazyme Biotech Co., Ltd.) and transformed into *Escherichia coli*. The recombinant plasmids were validated by Sanger sequencing, and 29 cDNA sequences that met the requirements were successfully screened.

### Probes labeling

An improved method was adopted for single-gene FISH probe labeling. The presence of repetitive sequences was checked by RepeatMasker (http://repeatmasker.org/cgi-bin/WEBRepeatMasker) in selected full-length cDNA sequences, and then specific primers for each probe were designed to avoid repetitive sequences in the cDNA sequences. cDNA fragments were amplified from the clones using specific primers. The PCR products were purified using the Omega E.Z.N.A.® MicroElute Cycle-Pure Kit (Cat. # D6293-02) and then digested with DNase I (Thermo Fisher Scientific Inc., Carlsbad, CA, USA Cat. # EN0525), a total of 25 μl enzyme system, containing 1–2 ug PCR products and 50 mU DNase I. The digestion time at 37 ℃ was approximately 2 h and was adjusted according to the length of PCR products. The digestion reaction was terminated by heating at 80 °C for 5 min. The final digestion product was approximately 50–500 bp. After digestion the products were labeled with fluorescein-12-dUTP (Roche Diagnostics GmbH, Mannheim, Germany Cat. # 11373242910) or tetramethyl-rhodamine-5-dUTP (Roche Diagnostics GmbH, Mannheim, Germany Cat. # 11534378910) using the random primer labeling method as described previously with minor modifications [20]. Briefly, the reaction time was extended to two days at 37 ℃ and the probes were purified using the Omega DNA Probe Purification Kit (Cat. # D6538-02) according to the manufacturer’s recommendations.

Four repetitive sequences were used as chromosomal markers: pSc119.2, 45S rDNA, pAs1, and (AAG)_10_. The designated oligonucleotides Oligo-pSc119.2–1 plus Oligo-pSc119.2–2 represent pSc119.2, pAs1-1 plus pAs1-2 and Oligo-pTa71-2 represent pAs1 and 45S rDNA, respectively [50]. Repetitive sequences pSc119.2 and (AAG)_10_ were end-labeled using 5(6)-carboxyfluorescein (5(6)-FAM; green), and 45S rDNA and pAs1 were end-labeled using 5-carboxy-tetramethylrhodamine (5-TAMRA; red) (Sangon Biotech Co., Ltd., Shanghai, China) to generate FISH probes.

Genomic DNAs of *P. stipifolia* (2*n* = 2*x* = 14; StSt) and *H. bogdanii* (2*n* = 2*x* = 14; HH) were fragmented by autoclaving and labeled with tetramethyl-rhodamine-5-dUTP (red) and fluorescein-12-dUTP (green) using a random primer labeling method as described by Dou et al. [20].

### Chromosome preparation

Seeds were germinated on moist filter paper in Petri dishes at room temperature. Root tips with a length of 1–2 cm were collected, pretreated with N_2_O at 7 atm for 2 h, and fixed in 3:1 (v/v) ethanol: glacial acetic acid. Chromosome spreading and slide preparation were performed as described by Xie et al. [19].

### FISH and GISH

FISH was performed as described previously [51, 52], with minor modifications. In the process of sequential FISH, a single-gene probe was used for the first round of hybridization. The second and third round hybridizations were carried out using the probe combination 45S rDNA and pSc119.2, and the combination of pAs1 and (AAG)_10_, respectively. The last round was conducted by genomic in situ hybridization (GISH) with genomic DNA probes of *P. stipifolia* and *H. bogdanii*. Moreover, a combination of several single-gene probes was used in the first round of hybridization to the remaining materials efficiently after confirmation of the distribution of single-gene probes in the chromosomes in materials DY and B05 of *E. sibiricus* and *E. nutans*, respectively.

### Microphotometry and chromosome measurements

Images were captured using a cooled charge coupled device camera (DP80) under a fluorescence microscope (Olympus BX63). Finally, images were adjusted with Adobe Photoshop 6.0 for contrast and background optimization. Chromosome measurements were carried out using karyotype software according to previous reports [28, 53].

## Supplementary Information

**Additional file 1: Fig. S1.** Prediction of CRs processes in homoeologous groups 2 of the three species. **Fig. S2.** Prediction of CRs processes in groups 4 and 5 of the three species.  **Fig. S3.** Molecular karyotype of sample DY of *E. sibiricus* with 59 single-gene probes and repetitive sequences probes. **Fig. S4.** Idiogram for chromosome collinearity of those with polymorphic CRs in *E. sibiricus*. **Fig. S5.** Molecular karyotype in sample PJC of *E. nutans* with 59 cDNA probes and repetitive sequences probes. **Fig. S6.** Idiogram for chromosome collinearity of those with polymorphic CRs in *E. nutans*.  **Fig. S7**. Chromosome variants of 11 materials in 3 species. 

**Additional file 2: Table S1.** The information of single-gene probes. **Table S2.** Localization of single-gene probes by FISH on chromosomes of *H. bogdanii*. **Table S3.** Localization of single-gene probes by FISH on chromosomes in material DY of *E. sibiricus*. **Table S4.** Localization of single probes by FISH on chromosomes in material PJC of *E. nutans*.

## Data Availability

The dataset generated during the current study are available in National Center for Biotechnology Information (NCBI) and the accession number is SRR22518291.

## Abbreviations

FISH: Fluorescence in situ hybridization
CRs: Chromosomal rearrangements
QTP: Qinghai-Tibet Plateau
GISH: Genomic in situ hybridization
PaI: Paracentric inversion
PeI: Pericentric inversion
RT: Reciprocal translocation
T: Translocation
Du: Duplication and insertion
De: Deletion
NCI: Nucleo-cytoplasmic interaction
ESTs: Expressed sequence tags
