# Supplementary material for: Single-gene FISH maps and major chromosomal rearrangements in Elymus sibiricus and E. nutans
Source: BMC Plant Biol. 2023 Feb 17;23:98. doi: 10.1186/s12870-023-04110-4 (PMC9936730; doi:10.1186/s12870-023-04110-4)
Supplement: Supplementary file 1 — Additional file 1: Fig. S1. Prediction of CRs processes in homoeologous groups 2 of the three species. Fig. S2. Prediction of CRs processes in groups 4 and 5 of the three species. Fig. S3. Molecular karyotype of sample DY of E. sibiricus with 59 single-gene probes and repetitive sequences probes. Fig. S4. Idiogram for chromosome collinearity of those with polymorphic CRs in E. sibiricus. Fig. S5. Molecular karyotype in sample PJC of E. nutans with 59 cDNA probes and repetitive sequences probes. Fig. S6. Idiogram for chromosome collinearity of those with polymorphic CRs in E. nutans. Fig. S7. Chromosome variants of 11 materials in 3 species. [file 12870_2023_4110_MOESM1_ESM.pptx]

## Slide 1
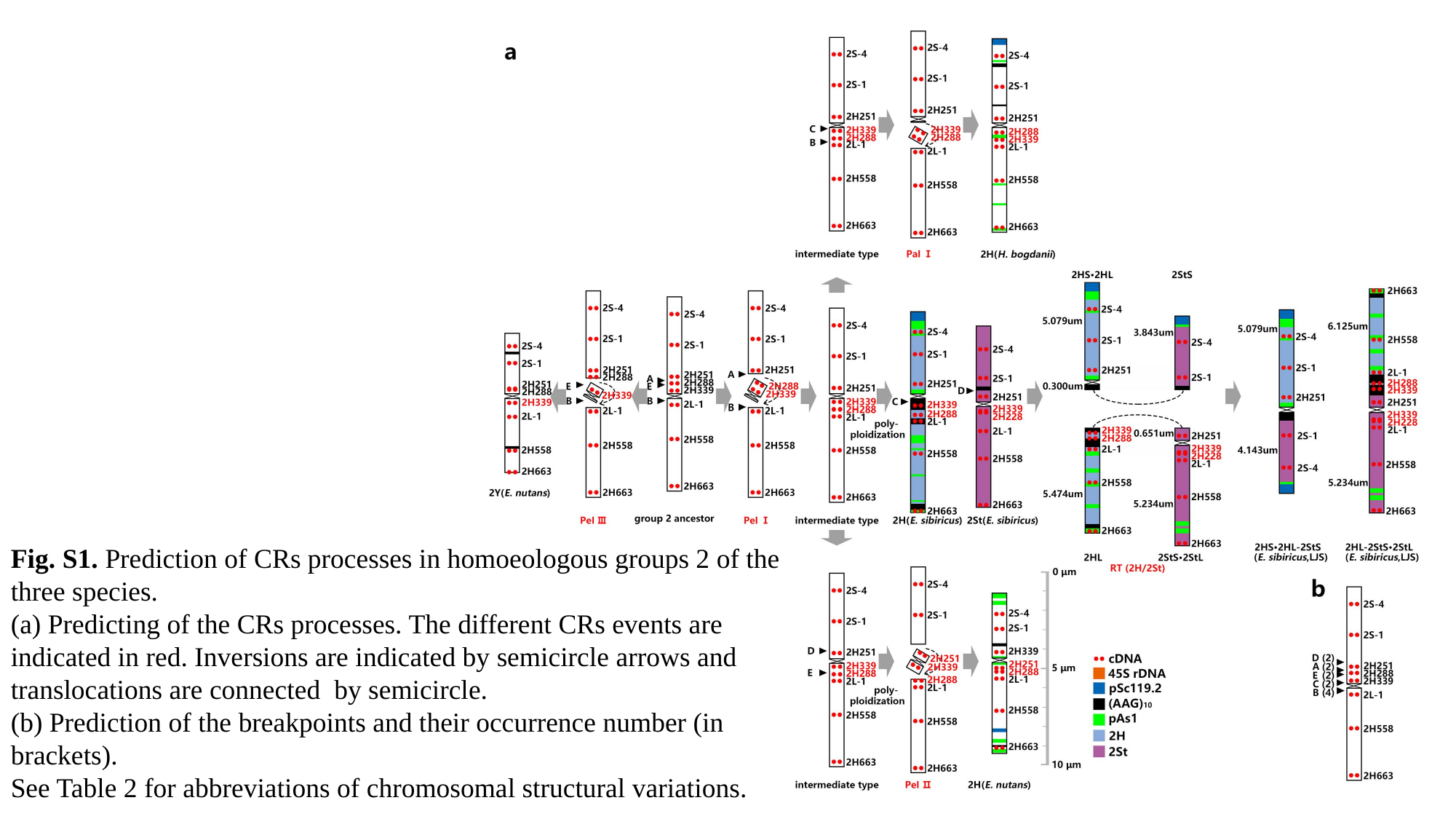

Fig. S1. Prediction of CRs processes in homoeologous groups 2 of the three species.
(a) Predicting of the CRs processes. The different CRs events are indicated in red. Inversions are indicated by semicircle arrows and translocations are connected by semicircle.
(b) Prediction of the breakpoints and their occurrence number (in brackets).
See Table 2 for abbreviations of chromosomal structural variations.

## Slide 2
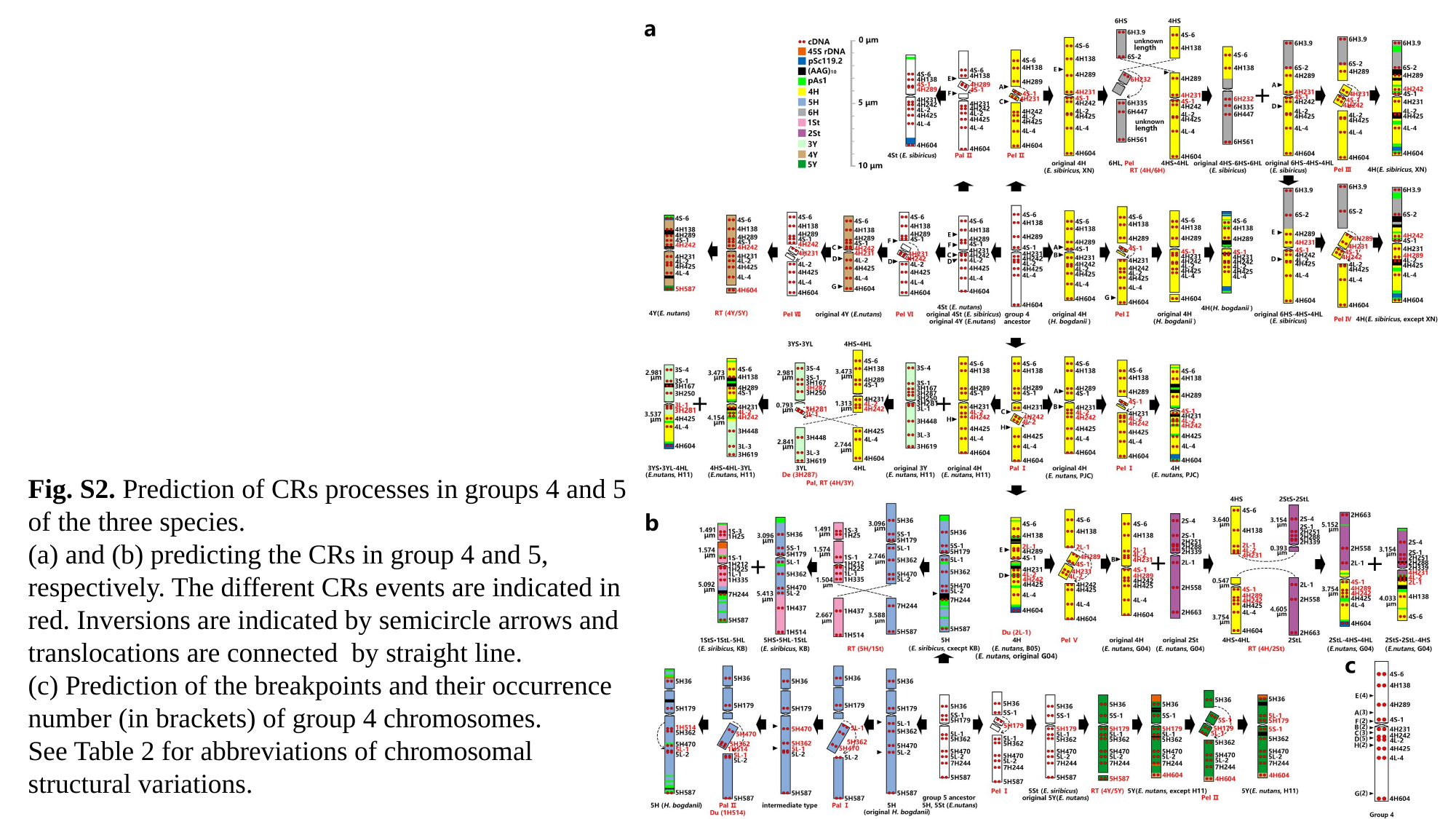

Fig. S2. Prediction of CRs processes in groups 4 and 5 of the three species.
(a) and (b) predicting the CRs in group 4 and 5, respectively. The different CRs events are indicated in red. Inversions are indicated by semicircle arrows and translocations are connected by straight line.
(c) Prediction of the breakpoints and their occurrence number (in brackets) of group 4 chromosomes.
See Table 2 for abbreviations of chromosomal structural variations.

## Slide 3
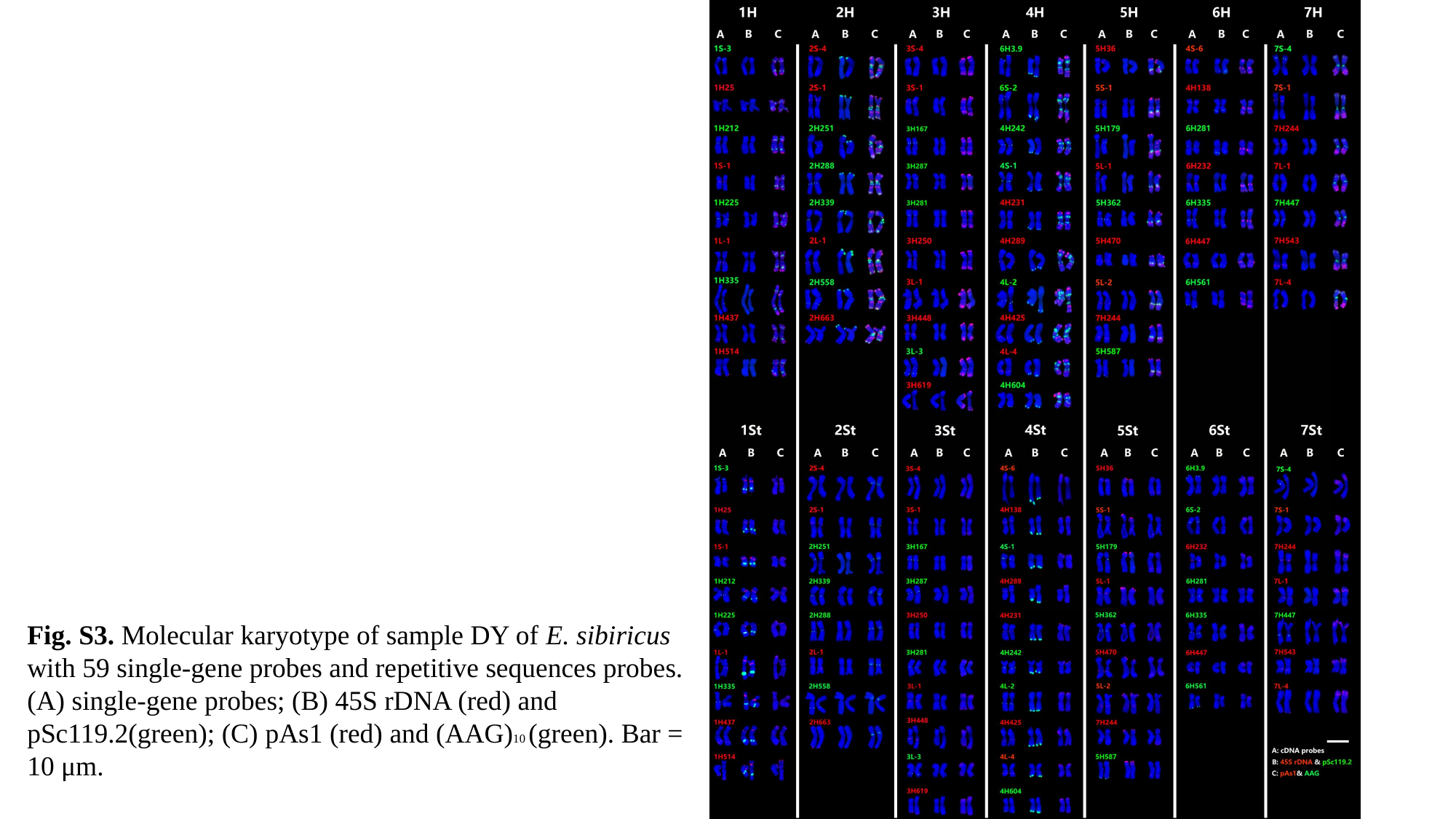

Fig. S3. Molecular karyotype of sample DY of E. sibiricus with 59 single-gene probes and repetitive sequences probes.
(A) single-gene probes; (B) 45S rDNA (red) and pSc119.2(green); (C) pAs1 (red) and (AAG)10 (green). Bar = 10 μm.

## Slide 4
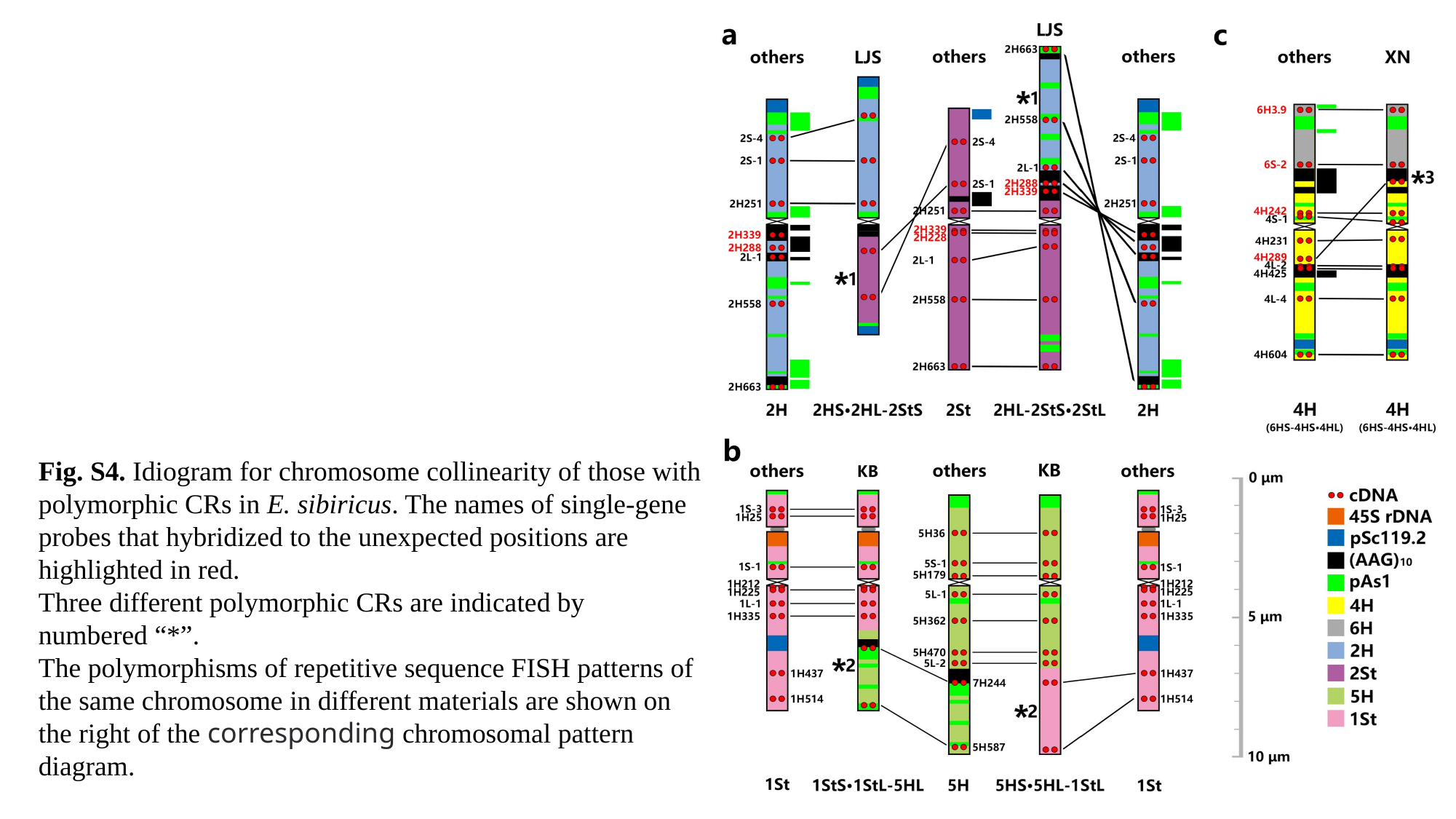

Fig. S4. Idiogram for chromosome collinearity of those with polymorphic CRs in E. sibiricus. The names of single-gene probes that hybridized to the unexpected positions are highlighted in red.
Three different polymorphic CRs are indicated by numbered “*”.
The polymorphisms of repetitive sequence FISH patterns of the same chromosome in different materials are shown on the right of the corresponding chromosomal pattern diagram.

## Slide 5
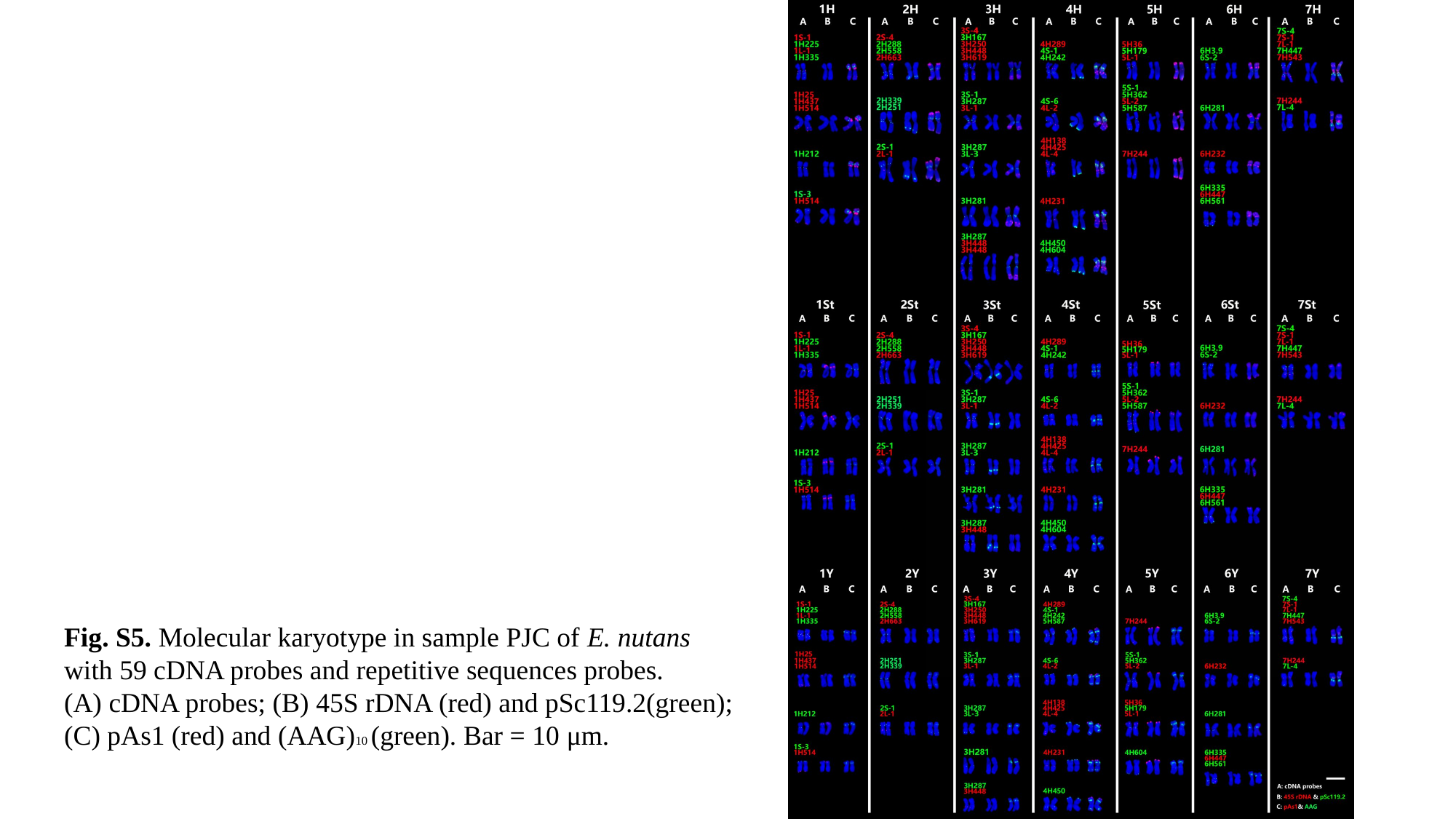

Fig. S5. Molecular karyotype in sample PJC of E. nutans with 59 cDNA probes and repetitive sequences probes.
(A) cDNA probes; (B) 45S rDNA (red) and pSc119.2(green); (C) pAs1 (red) and (AAG)10 (green). Bar = 10 μm.

## Slide 6
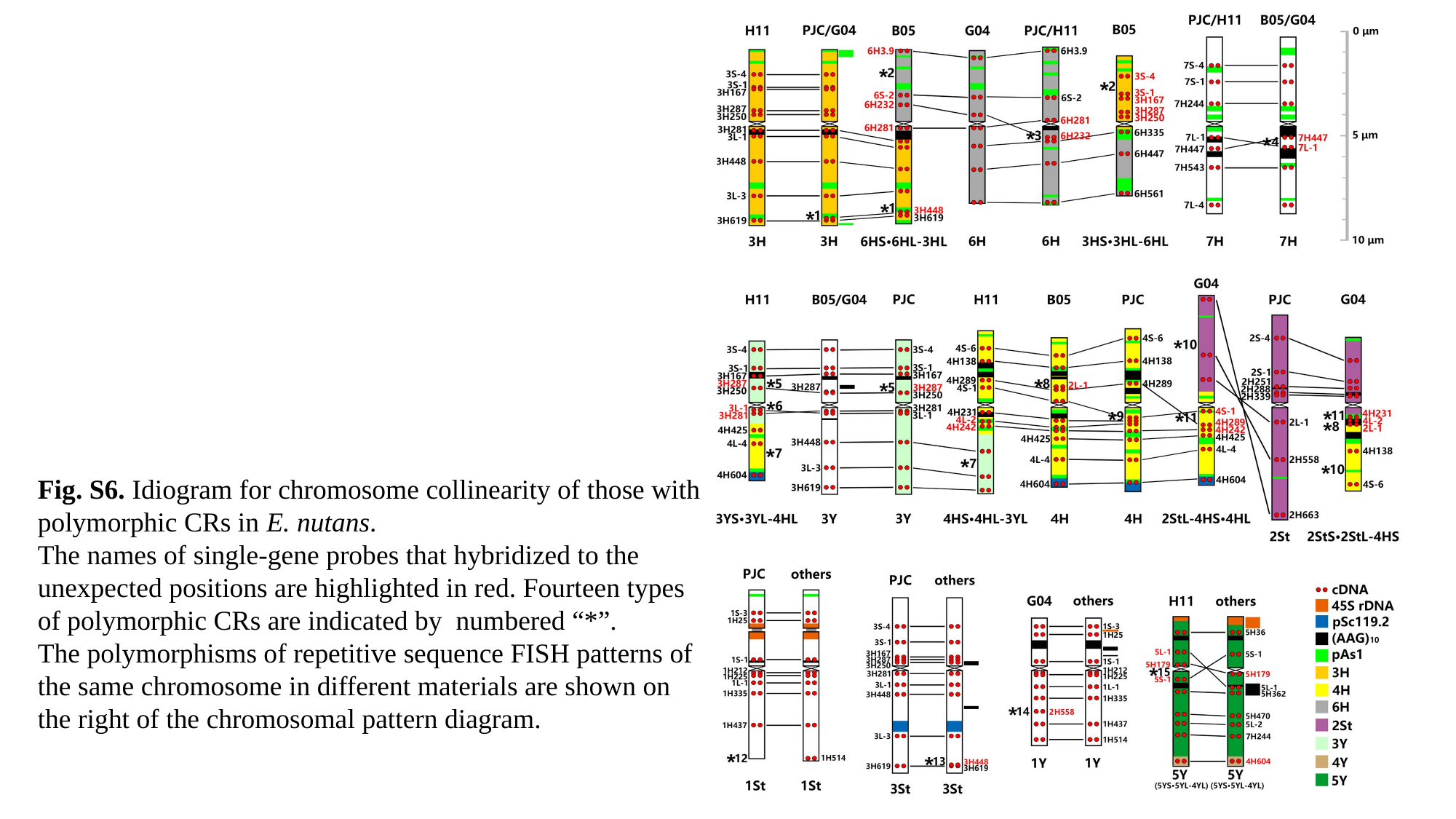

Fig. S6. Idiogram for chromosome collinearity of those with polymorphic CRs in E. nutans.
The names of single-gene probes that hybridized to the unexpected positions are highlighted in red. Fourteen types of polymorphic CRs are indicated by numbered “*”.
The polymorphisms of repetitive sequence FISH patterns of the same chromosome in different materials are shown on the right of the chromosomal pattern diagram.

## Slide 7
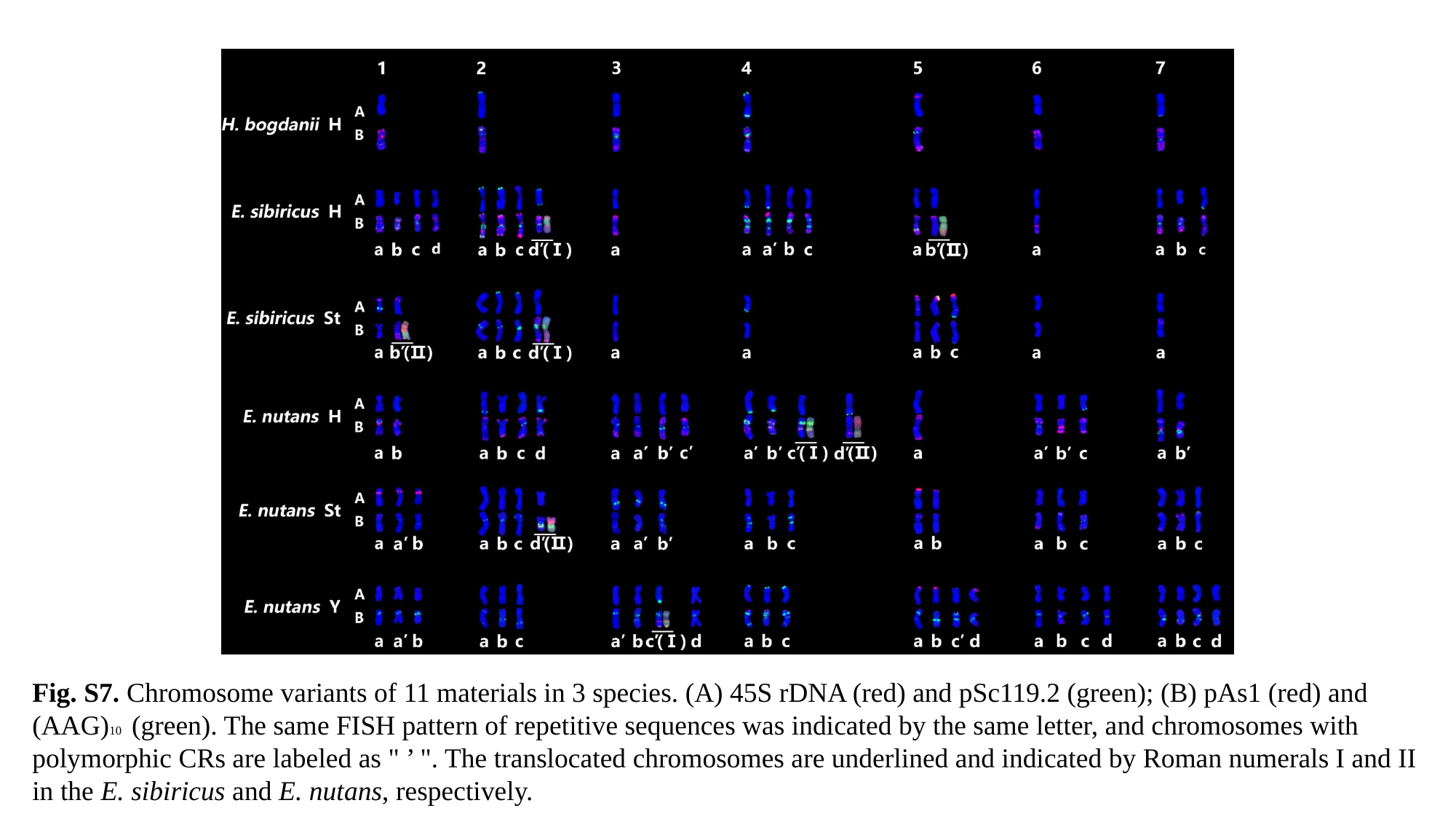

Fig. S7. Chromosome variants of 11 materials in 3 species. (A) 45S rDNA (red) and pSc119.2 (green); (B) pAs1 (red) and (AAG)10 (green). The same FISH pattern of repetitive sequences was indicated by the same letter, and chromosomes with polymorphic CRs are labeled as " ’ ". The translocated chromosomes are underlined and indicated by Roman numerals I and II in the E. sibiricus and E. nutans, respectively.
